# Temperature in Croup

**Published:** 1878-04

**Authors:** 


The Temperature in Croup.—It has been maintained, and that by the best authorities, that true croup can be differentiated from false or catarrhal croup simply by using the thermometer, in that "in most cases catarrh of the larynx runs its course without fever whilst true croup is accompanied by high fever" (Niemeyer). Dr. M. Loeb (*Jahrbuch f. Kinderheilkunde*, XII., 1 and 2, 1877) states that in two cases he saw true croup run its course without any fever whatever, and relates the following case which is of interest in more than one respect. The patient was a girl, aged 2¼ years. When first examined there was present only mucous and sub-mucous râles over both sides of the chest and slight redness of the fauces. The temperature in the rectum was normal. The cough was neither rough nor barking, therefore bronchial catarrh had to be diagnosticated. On the same day, in the evening, the whole condition had changed; great dyspnœa presented itself, the cough had changed, the epigastrium was drawn in with every inspiration. "Under such circumstances diagnosis could hardly be doubtful." Emetics, inunctions of mercurial ointment and cold applications to the neck were ordered. Notwithstanding copious emesis the whole condition did not change for the better. Cyanosis became more marked, dyspnœa greater and yet the temperature did not rise. Tracheotomy was indicated which, after several hours had elapsed, was also performed. "Unfortunately the large middle lobe of the thyroid gland caused so much hemorrhage that the child, already nearly dead, expired upon the table." Post-mortem examination revealed croupous exudation upon the mucous membrane of the larynx, this extended down to the bifurcation of the trachea, the fauces were free from diphtheritic exudation. "It therefore must be claimed, both in regard to diagnosis and prognosis, that individual cases of croup may run their course without losing their malignant character, without any fever whatsoever." On the other hand, cases of catarrhal croup have frequently been observed in which the fever was very high. In connection with emetics the author has a foot-note regarding the action of apomorphia, which has offered no advantages over the ordinary emetics in use, in fact refusing to act in cases where sulphate of copper produces abundant emesis.—*The Clinic.*

